# Cigarette Smoking among Adolescents in Northwest Ohio: Correlates of Prevalence and Age at Onset

**DOI:** 10.3390/ijerph5040278

**Published:** 2008-12-15

**Authors:** Sadik A. Khuder, James H. Price, Timothy Jordan, Saja S. Khuder, Kathi Silvestri

**Affiliations:** 1 Department of Medicine, University of Toledo, 3120 Glendale Avenue, Toledo, OH 43614, USA; E-mail: Saja.Khuder@utoledo.edu (S. S. K); 2 Department of Health & Rehabilitative Services, University of Toledo, Mail Stop # 119, Toledo, OH 43606, USA; E-Mails: Jprice@utnet.utoledo.edu (J. P.); Tjordan2@utnet.utoledo.edu (T. J.); 3 Hospital Council of Northwest Ohio, 3231 Central Park West Drive, Suite 200, Toledo, OH 43617, USA; E-mail: ksilverstri@hcno.org (K. S.)

**Keywords:** Cigarette smoking, adolescent, predictors, smoking initiation

## Abstract

This study examined the prevalence and correlates of smoking initiation among adolescents. We have used data from adolescents (n=5,392) ages 10–18 who participated in the 2003 Tobacco Survey, a representative sample of adolescents in Northwest Ohio. A self-report of cigarette smoking was obtained using a questionnaire administered in classrooms. Data were analyzed using weighted chi-square and multiple logistic regressions in SAS that accounted for the survey design. The prevalence rates for adolescents that ever tried smoking were 7.4% in elementary (grades 4–5); 17.7% in middle (grades 6–8), and 41.4% in high (grades 9–12) schools, respectively. The highest prevalence rate was among Hispanics. Having a close friend that smoked and a smoker at home correlated significantly with both initiation of smoking and smoking at an earlier age. Smoking was correlated with low academic achievement among adolescents in all grades. Students who reported smoking by parents or siblings were significantly more likely to start smoking at an earlier age, compared to other students living in a non-smoking home environment. Smoking prevention program should include components focused on adolescents’ home environment and should start as early as the 4^th^ grade.

## 1. Introduction

Tobacco is the leading cause of preventable morbidity and mortality worldwide. In the United States, during 1997–2001, cigarette smoking and exposure to tobacco smoke resulted in an estimated 438,000 premature deaths, and 92 billion dollars in annual productivity losses [1]. Cigarette smoking is prevalent in Ohio and the state ranks eighth in the nation in smoking rate. The current smoking prevalence rate in 2003 was 25.4% among adults [2] and was 22.2% among grades 9–12 [3].

Two of the national health objectives for 2010 are to reduce the prevalence of current tobacco use to ≤21% among adults and to ≤16% among high school students [4]. An important step toward achieving these objectives is to address the issue of smoking initiation in adolescents [5].

Tobacco smoking among adolescents is of public concern because of the immediate and long-term adverse health consequences such as asthma, chronic cough, cancers, chronic obstructive airways and cardiovascular diseases. Regular smoking by children is associated with increased risk of new-onset asthma [6]. Adolescent smokers are at a higher risk for developing cardiovascular disease later in life, compared to their non-smoking counterparts [7].

Smoking initiation in adolescents is also a problem with great impact on individual’s well being. It may lead to retardation in physical development [8]. Adolescents who smoke are more likely to use illicit drugs and alcohol and be involved in deviant behavior [9]. Smoking occurs within the context of other risk-taking and self-destructive behavior among both children and older adolescents [10].

The age at which the individual begins smoking may influence his/her health at an older age. Children who begin to smoke during elementary school may be more likely to smoke as adults than individuals who begin at older age [11]. Those who started smoking before 16 years of age had twice the odds of not quitting smoking compared to those who started at a later age [12].

To determine the interventions needed to prevent early initiation of smoking, it is important to identify predictors of tobacco use by adolescents. Many variables that influence the likelihood of smoking initiation in adolescent population have been studied. However, the effect of these variables on age at onset is not well understood. In this paper, we investigated predictors of smoking initiation and age at start of smoking among adolescent in Northwest Ohio.

## 2. Methods

The Northwest Ohio Youth Tobacco Survey (NOYTS) conducted in 2003 was a cross sectional study, using a multi-stage cluster sampling technique. In the first stage, schools were selected based on agreement to participate in the survey. In the second stage, systematic samples of classes were selected. All students in the selected classes were eligible to participate in the survey.

All schools (elementary, middle, and high schools) in Northwest Ohio were asked to participate in the survey. A total of 124 schools (64%) out of 194 agreed to participate and to provide access to their students. These schools were representative of all the 16 counties in Northwest Ohio.

The survey questionnaire was developed based on the 2001–2002 National Youth Tobacco Survey (NYTS) core questionnaire [13]. The NYTS questionnaire was modified to include questions needed in the project for youth tobacco program in Northwest Ohio. Additional questions were added mainly regarding the family role and potential effect of family member on tobacco use by their children. The final study questionnaire consisted of 77 multiple-choice questions that included demographic information, individual smoking habits, exposure to tobacco advertisements, attitudes and beliefs towards tobacco, exposure to passive smoking, access to tobacco products, school anti-smoking and prevention programs, and habits and attitudes of family and friends regarding tobacco use.

Ever smokers were identified according to the question “Have you ever smoked cigarettes daily, that is, at least one cigarette every day for 30 days?” Subjects were considered smokers if they answered “yes”. Smoking status was assessed by a question asking to describe their smoking at the time of the survey. Subjects were considered current smokers if they answered “I smoke” or “I quit, but now I smoke again. Subjects were considered past smokers if they answered “I quit smoking within the past 6 months or more than 6 months ago”. Age at smoking onset was obtained from the question “How old were you when you smoked a whole cigarette for the first time?” Age at start of smoking was defined using 6 ordered categories (“8 years old or younger”, “9 or 10”, “11 or 12”, “13 or 14”, “15 or 16”, and “17 years old or older”).

Researchers at Great Lakes Marketing (GLM), a commercial company, implemented the survey and supervised the data collection procedures. They contacted school administrators to obtain permission to survey students and also were involved in the design of the survey. The survey questionnaire was tested in a pilot study involving five classrooms from the selected schools. Students required an average of 30 minutes to complete the survey. Data were collected at schools, using a self administered questionnaire. The teacher left the classroom after introducing the researcher, who explained that students did not have to write their names on the questionnaires and assured strict confidentiality of the responses. Subsequently, the questionnaires were distributed in the class by the researcher.

Data were weighted to provide estimates for adolescents in Northwest Ohio. For the estimation of prevalence or proportions, the weights were selected to account for design effect (selection of school and class levels), non response and the sample representation relative to racial and gender distribution in the total population. To some extent, the weighted estimates were representative of the population of adolescents aged 10 to 18 living in Northwest Ohio in 2003.

Exploratory analyses were used to investigate the distribution of the independent variables. Categorical variables were described as percentages together with their corresponding 95% confidence intervals and quantitative variables were described as mean (SD). Binary logistic regression was used to examine initiations of smoking as it relates to some established risk factors (age, gender, race, living with smoker, grades and number of closest friends who smoke cigarettes). Adjusted odds ratio (OR) along with 95% confidence interval (CI) was calculated for the risk factors. Generalized ordered logistic regression was used to identify predictors of age at start of smoking. This analysis uses a cumulative logit model with the proportional odds assumption. Age at onset of smoking was recorded using 6 ordered categories. All the analysis was done using SAS 9.1 software. Weighted analysis was performed using both the suveyfreq and surveylogistic procedures in SAS [14]. All statistical tests reported are two-sided, and a p-value < 0.05 is considered to be statistically significant.

## 3. Results

Of the original sample of the students who participated in the study (n=5,392), a weighted sample was estimated as 5,021. About 54% were males; 87% whites and with almost equal distribution from grade 4 to grade 12. Approximately 26% of the population has initiated tobacco smoking. The distribution of smoking initiation by weighted demographic characteristics of the study population is shown in Table 1.

Smoking prevalence among males was 26.8%, compared to 25.6% among females and the difference is not statistically significant. The percentage of Hispanic smokers was significantly higher than other races. Smoking prevalence was 6.4% among 4^th^ graders and was 50.7% among 12th grade students. Smoking prevalence increased with age, and increased sharply betweens grades 8 and 9. About 7.4% of the students in elementary school (grades 4–5) were ever smokers compared to 17.7% in middle school (grades 6–8) and 41.4% in high school (grades 9–12) (Table 2).

The rate of smoking initiation in males was similar to that for females in all grades. The smoking prevalence was significantly different by race in grades 6–8, and grades 9–12. The highest initiation rate was among Hispanics. Initiation rates among African American were not statistically different from the rates among whites. Smoking was significantly correlated with lower academic achievement and for all grade levels. The percentage of ever smokers among students with bad performance at school was 13.2% for grades 4–5, 34.5% for grades 6–8, and was 55.2% for grades 9–12. Living with a smoker was associated with higher percentage of ever smokers for all grades. Peer smoking was significantly correlated with higher prevalence of smoking in all grades. When none of the closest friends smoked, smoking prevalence rates were 4.5% for grades 4–5, 9.6% for grade 6–8, and 26.3% for grades 9–12.

Table 3 presents results of logistic regression model identifying predictors of smoking initiation. The OR for smoking initiation was increased as a function of grade level. Compared to grade 4–5, the OR for smoking initiation increased from 2.15 (CI, 1.64–2.81) for grade 6–8 to 6.22 (CI, 4.79–8.08) for grade 9–12.

No significant difference was found between males and females. Hispanic students were one and half times more likely to initiate smoking than Whites (p=0.04). Student with poor academic achievement was three times more likely to initiate smoking compared to an “A” student. Parent smoking was associated with increased risk of initiating smoking. The OR for a mother smoker was higher than the OR for a father smoker. In the same household, brother or sister smoking was the strongest correlate of smoking initiation. Having one closest friend smoking increased the likelihood of initiating smoking by almost five times.

Table 4 presents results of ordinal logistic regression model for correlates of age at initiation of smoking. Race was a significant correlate of earlier age at initiation of smoking. Hispanic or others were two times more likely to initiate smoking at earlier age compared to Whites or African American. Living with a smoker significantly increased the likelihood of initiating smoking at an earlier age.

Participants reporting having a best friend who smokes were three times more likely to start smoking at earlier age. Participants having a higher percentage of friends who smoke were four times more likely to initiate smoking earlier than other students. Academic achievement was a significant predictor of early age at smoking initiation. Participants who made B or C were more likely to initiate smoking earlier compared to those making A. Similarly those who made D or F had three time the odds of reporting initiation of smoking at earlier age compared to those making A (OR=2.90; p=0.0001).

## 4. Discussion

This study examined the prevalence and correlates of smoking initiation among adolescents in Northwest Ohio. The estimated prevalence rates are comparable to those reported in the 2003 Youth risk behavior surveillance [3].

We found that gender is not a significant correlate of either smoking initiation or age at the start of smoking. This is in line with the finding of other studies [15]. This is also contrary to other studies in the literature. Males were more likely to initiate daily smoking than females from age 13 to age 21 as reported by Hill *et al.*[16]. By age 15 more girls than boys reported smoking [17]. Higher prevalence of smoking among males compared to females has been reported in a cohort of Brazilian adolescent aged 10–12 years [18].

In contrast to national data for youths aged 12–17 [19], prevalence rates of smoking were higher among Hispanics than Whites, African American and other. However, this finding is consistent with previous study indicating that higher level of acculturation predicts increased smoking among Hispanic adolescents [20]. Current smoking rates for Hispanic high school students in 2007 were higher than smoking rates for African-American students, but lower than the rates of White students. [21].

No significant difference was found between Whites and African American adolescents in our study. Two studies in the literature [22, 23] have shown that African-American adolescents are less likely to smoke cigarettes than White youth. The national data suggest that this pattern changes in late adolescence and early adulthood. Specifically, African-American adults have a relatively high smoking prevalence rate when compared with other racial/ethnic groups. It is possible that African American in grades 9–12 underreported their cigarette use compared to Hispanic students. Two studies in the literature concluded that African American adolescents [24] and young adults [25] underreported cigarette use to a greater extent than Whites.

Smoking by a family member is a strong correlate of smoking. Parent smoking habits was significantly related to both initiation and early onset of smoking. The association between exposure to smoking parents and smoking status of a teenager is consistent with other studies [26, 27]. Parent smoking contributes to the onset of daily smoking in their teenagers even if parents practice good family management, hold norms against teen tobacco use, and do not involve their children in their own tobacco use [16].

In this study, the likelihood of initiating smoking when mother smoked increased two fold and this tended to occur at younger age. Mothers play a crucial role in molding the behavior of young children. The finding that mother’s smoking correlates with earlier age at onset is important and point to the triggering effect earlier in life. Mothers’ effects on adolescent cigarette smoking are mostly during earlier age and thus most of the initiation triggered by mother smoking will occur earlier in life. Offspring of nicotine-dependent women had a higher rate of nicotine dependence, and this relation is greater if the mother also smoked during the offspring’s gestation [28]. Our finding here is important as a large number of females smoke and smoking by pregnant women is a problem in Northwest Ohio.

Smoking by a sibling tripled the likelihood of both initiation and early onset of smoking. Sibling influence could be through social influence affecting the adolescent’s thoughts, feeling, and actions. The social influence of families and other significant individuals is well supported in the literature [29].

Having closest friends smoking was correlated with age at smoking initiation. Those who started smoking earlier tend to be triggered by a friend smoking. It is possible that those who started earlier accumulated more friends with similar smoking habit over a longer time than those who started later. Another explanation for this may be the tendency of smokers to associate with smokers. However, peer smoking appears to be the most important factor influencing smoking initiation. Some studies [30, 31] suggested that susceptibility to peer pressure is influenced by race and gender. The competing influences such as peer smoking and mother’s smoking play an important role in adolescent smoking behavior. One review concluded that smoking behavior of family and friends was consistently associated with adolescent smoking [32]. Thus children living with smokers may be not only at high risk of initiating smoking, but also of smoking at a very young age. Peer influence may modify the association between parent and adolescent cigarette smoking. The association between parent and adolescent cigarette smoking may weaken as adolescents age [33], or peer influence may increase as adolescents age while the influence of parents remains generally the same [34].

Academic achievement was a significant correlate of smoking initiation. Failing grades was correlated with increased risk of smoking initiation and this was more pronounced in grades 6–8. This finding is consistent with a number of different studies in the literature. [10, 35–36]. Performance at school was also correlated with earlier age at onset of smoking. Those with low grades (D or F) tend to initiate smoking earlier than other students with higher grades. This finding is new and point to the need to educate children with bad performance in elementary school about the danger of initiating smoking. However, due to the nature of the data, we cannot ascertain the directionality of the cause-effect relation, if any, which may explain this correlation. For example smoking may lead to absence from classes, less attention and thus students may perform poorly at school. On the other hand, poor performance may by itself lead the student to be associated with other students with poor performance and this may ultimately result in initiation of smoking.

The present study has some limitations. First, conclusions that can be drawn from this study are limited by the cross-sectional nature of the data. Specifically, it is not possible to determine whether the observed differences (for example, by school performance) emerged before or after smoking initiation. We could use the same argument to explain the correlation between closest friend smoking and onset of individual smoking. Second, the sample size was relatively small for Hispanic and other races to allow for additional analyses stratified by race. Third, we did not investigate personal factors (such as depressive symptoms, weight control, and pocket money) in this study and these factors may affect smoking initiation in adolescents. For example, adolescents with depressive symptoms are more likely to start smoking compared to non-depressed adolescent [37]. Younger children may smoke to cope with anxiety and depression [10]. In addition, smoking and depression could be common consequences of familial and other factors. [38]. Coogan *et al*. [10] reported an association between dieting and smoking among girls of all ages, which suggest that young girls may also smoke to control their weight as early as elementary schools. Pocket money is also positively associated with smoking initiation in adolescent. [39]

Finally, smoking status was based on self-report and therefore subject to respondent recall and may be deliberate misreporting. Data on current smoking were not verified by biomarkers such as cotinine assessment or exhaled carbon monoxide. However, we used a validated NYTS methodology, and weighted analysis that adjusted for design effect and non-response bias. By doing so, our findings are valid, and could be compared to other studies using the same methodology. High school students in the United States report personal health risk behaviors reliably over time as reported by Brener *et al.*[40].

In conclusion, our data indicated that race, smoking by family member or closest friend, and performances in school are significant correlates of smoking initiation in adolescents. These variables are important not only in affecting experimentation with cigarette smoking but also in facilitating this at an earlier age. In order to reduce the risk of smoking initiation, smoking prevention programs need to focus on environmental influences of early adolescent smoking such as parent and peer smoking. Smoking prevention programs should include components focused on adolescents’ home environment and to start as early as the 4^th^ grade. The possible impact of the smoking ban in Ohio in reducing cigarette smoking among adolescents in Northwest Ohio remains to be observed.

## Figures and Tables

**Table 1:** The distribution of smoking initiation by gender, race and grade level.

**Factors**	**Weighted Total**		**Ever Smoked**		**p-value**

	**N**	**%**	**N**	**%**	

**Gender**					0.332

Male	2,689	53.6	721	26.8	
Females	2,332	46.4	597	25.6	

**Race**					0.0001
	
Whites	4,342	86.7	1,099	25.3	
Blacks	376	7.5	102	27.1	
Hispanic	220	4.4	94	42.7	
Other	71	1.4	21	29.6	

**Grade**					0.0001

4	549	10.9	35	6.4	
5	541	10.8	46	8.5	
6	541	10.8	59	10.9	
7	560	11.1	109	19.5	
8	549	10.9	123	22.4	
9	636	12.7	202	31.8	
10	547	10.9	224	41.0	
11	548	10.9	240	43.8	
12	550	11.0	279	50.7	

All	5,021	100.0	1,318	26.2	

**Table 2:** The distribution of ever smokers by demographics for different grade levels.

	**Grade 4–5**		**Grade 6–8**		**Grade 9–12**	

	%ever	p-value	%ever	p-value	%ever	p-value

**Gender**						
Male	8.0	0.367	17.4	0.733	42.7	0.455
Females	6.5		18.0		40.6	

**Race**						
Whites	7.2	0.099	16.2	0.0001	41.1	0.015
Blacks	4.7		26.8		36.3	
Hispanic	17.2		30.4		53.8	
Other	6.1		17.8		41.4	

**Academic achievement**						
A	2.7	0.0001	8.5	0.0001	24.1	0.0001
B or C	7.4		15.9		44.9	
D or F	13.2		34.5		55.2	

**Living with a smoker**						
None	3.4	0.0001	8.2	0.0001	32.5	0.0001
Mother	14.1		31.1		51.9	
Father	8.8		18.5		40.4	
Brother or sister	24.3		37.3		64.3	
Other	13.1		28.9		52.0	

**Closest friend smoke**						
None	4.5	0.0001	9.6	0.0001	26.3	0.0001
One	26.5		34.4		48.7	
Two or more	30.9		42.3		61.6	

**Table 3:** Correlates of smoking initiation among adolescents in Northwest Ohio.

**Variable**	**OR**	**95% confidence interval**	**p-value**

**Grade**			
4–5	ref.		
6–8	2.15	1.64–2.81	0.0001
9–12	6.22	4.79–8.08	0.0001

**Gender**			
Female	ref.		
Male	1.04	0.90–1.20	0.608

**Race**			
White	ref.		
African American	0.90	0.68–1.19	0.449
Hispanic	1.41	1.01–1.97	0.041
Other	0.88	0.47–1.65	0.689

**Academic**			
**achievement** A	ref.		
B or C	1.69	1.39–2.05	0.0001
D or F	3.02	2.38–3.83	0.0001

**Living with a smoker**			
None	ref.		
Mother	1.90	1.58–2.29	0.0001
Father	1.35	1.07–1.70	0.01
Brother or sister	2.96	2.16–4.06	0.0001
Other	1.70	1.17–2.47	0.005

**Closest friend smoke**			
None	ref.		
One	2.94	2.39–3.61	0.0001
Two or more	4.90	4.13–5.81	0.0001

**Table 4:** Correlates of early initiation of smoking among adolescents in Northwest Ohio.

**Variable**	**OR**	**95% confidence interval**	**p-value**

**Race**			
White	1	-	-
Black	1.08	0.84–1.40	0.542
Hispanic	1.91	1.41–2.60	0.001
Other	2.00	1.13–3.54	0.018

**Living with a smoker**			
No smoker	1	-	-
Mother	2.07	1.73–2.48	0.001
Father	1.39	1.10–1.76	0.006
Brother or sister	2.88	2.17–3.83	0.001
Other	1.68	1.19–2.38	0.003

**Closest friend smoke**			
None	1	-	-
One	2.93	2.36–3.63	0.001
2–4	4.14	3.50–4.92	0.001

**Academic**			
**achievement** A	1	-	-
B–C	1.59	1.32–1.93	0.001
D–F	2.90	2.19–3.85	0.001
